# Impact of Bariatric Surgery on Hypoglossal Nerve Stimulation Outcomes

**DOI:** 10.1002/lary.70193

**Published:** 2025-10-14

**Authors:** Praneet C. Kaki, Troy Wesson, Alison Choi, Sophia Shah, Thomas Kaffenberger, Ryan Soose, Noah Parker, Maurits Boon, Colin Huntley, Chihun Jim Han

**Affiliations:** ^1^ Sidney Kimmel Medical College, Thomas Jefferson University Philadelphia Pennsylvania USA; ^2^ Department of Otolaryngology‐Head & Neck Surgery Indiana University School of Medicine Indianapolis Indiana USA; ^3^ University of Pittsburgh School of Medicine Pittsburgh Pennsylvania USA; ^4^ Department of Otolaryngology‐Head & Neck Surgery University of Pittsburgh Pittsburgh Pennsylvania USA; ^5^ Veterans Affairs Pittsburgh Healthcare System Pittsburgh Pennsylvania USA; ^6^ Department of Otolaryngology & Sleep Medicine Thomas Jefferson University Hospital Philadelphia Pennsylvania USA; ^7^ Department of Head & Neck Surgery Robert Wood Johnson Medical School New Brunswick NJ United States

**Keywords:** bariatric surgery, clinical, obesity, obstructive sleep apnea, sleep apnea, sleep medicine

## Abstract

**Objective:**

The effectiveness of hypoglossal nerve stimulation (HGNS) for residual obstructive sleep apnea (OSA) in patients with prior bariatric surgery (BS) has not been previously reported. We evaluate and compare HGNS outcomes in this unique population.

**Methods:**

We conducted a multi‐institutional retrospective review between 2014 and 2023. Patients with prior BS were compared to those without (nBS). A 1:2 propensity score matching (PSM) was performed using age, gender, race, and baseline BMI. Data collected included demographics, pre/post‐operative apnea‐hypopnea index (AHI), body mass index (BMI), and Epworth Sleepiness Score (ESS). Treatment success was defined using Sher15 criteria. Analyses were conducted in R‐Studio.

**Results:**

After PSM, 72 patients met inclusion (mean age 61.3 years, 62% male, 83% White), including 24 (33%) with BS. Sher15 response rates were 71% in BS vs. 56% in nBS (*p* = 0.2). BS patients had significantly greater mean AHI reductions than NBS (−28.44 ± 16.39 vs. −17.68 ± 21.23; *p* = 0.009). Both groups had comparable reductions in ESS (−4.67 vs. −3.44; *p* = 0.2), with a mean postoperative ESS of 6.78 in BS and 6.17 in NBS (*p* = 0.5). On multivariable linear regression adjusting for demographics, baseline AHI, and BMI, prior BS was independently associated with greater AHI reduction (β = −8.6; 95% CI −16, −1.1; *p* = 0.026).

**Conclusion:**

Patients with prior BS achieve comparable or superior outcomes following HGNS. Prior weight loss may enhance HGNS effectiveness and merits further study.

**Level of Evidence:**

3.

## 1 Introduction

Obstructive sleep apnea (OSA) is a common upper airway disorder associated with recurrent nocturnal oxygen desaturations and sleep fragmentation that affects nearly 1 billion individuals worldwide [1]. If left untreated, moderate to severe OSA has been linked with serious cardiovascular complications such as high blood pressure, heart disease, irregular heart rhythms, and stroke [2]. Obesity is a significant and well‐known modifiable risk factor for various conditions, including OSA, with each unit increase in body‐mass index (BMI) being associated with a 1.14× increased risk of developing OSA [3, 4, 5, 6, 7, 8].

Bariatric surgery has been proven to be an effective method in achieving significant long‐term weight loss and reducing associated long‐term medical comorbidities [9, 10]. Commonly performed procedures include sleeve gastrectomy, laparoscopic gastric banding, and Roux‐en‐Y Gastric Bypass (RYGB) [9, 10]. The beneficial impact of bariatric surgery on OSA severity is highlighted by a meta‐analysis reporting a notable average reduction in AHI of 38.2 events/h in patients who underwent bariatric surgery [11]. Another systematic review demonstrated that bariatric surgery improved OSA disease severity in 79% of patients, irrespective of the procedure type. Moreover, bariatric surgery has also demonstrated effectiveness in alleviating daytime sleepiness associated with OSA as measured by the Epworth sleepiness scale (ESS) [12]. Despite these significant changes in weight, AHI, and OSA‐related symptoms, a previous study by de Raaff et al. demonstrated that approximately a quarter of bariatric patients with severe OSA continue to have persistent moderate to severe OSA following their procedure [13]. Likewise, a meta‐analysis by Greenburg et al. reported residual moderate to severe OSA (AHI > 15 events/h) in up to 62% of patients after bariatric surgery [11, 13].

Continuous Positive Airway Pressure (CPAP) has traditionally been considered the first‐line treatment for OSA and in the setting of residual sleep apnea after bariatric surgery to help manage residual symptoms and manage cardiovascular risk correlated with moderate‐to‐severe OSA. Despite its effectiveness when utilized as intended, many patients are unable to tolerate it, with limited adherence ranging from 30% to 60% across different studies [14, 15, 16]. For select patients with obstructive sleep apnea (OSA) who have been intolerant to CPAP, hypoglossal nerve stimulation (HGNS) is an effective device‐based therapy that works by stimulating the hypoglossal nerve and opening the airway at multiple anatomic subsites [17, 18]. However, there is a lack of literature on the efficacy of HGNS in patients who have undergone prior bariatric surgery and have residual OSA. In this study, we compare the outcomes of HGNS in managing moderate to severe OSA among patients who have and have not undergone prior bariatric surgery. We hypothesize that prior bariatric surgery and associated weight loss may be an important precursor to improving the effectiveness of adjuvant HGNS.

## 2 Methods

This study was approved by the institutional review boards at Thomas Jefferson University Hospital, Indiana University Health University Hospital, and University of Pittsburgh Medical Centers, and was conducted under a collaborative research agreement among the three institutions.

### 2.1 Cohort Selection

A retrospective review of electronic medical records (EMR) was performed to identify patients who underwent HGNS (Inspire Medical Systems, Minneapolis, MN, USA) for CPAP‐intolerant OSA between 2014 and 2023 at three tertiary care facilities. Surgical intervention was preceded and guided by drug‐induced sleep endoscopy (DISE) for all patients [19]. Chart review was performed to identify patients who had undergone bariatric surgery prior to sleep surgery; the included bariatric procedures were Gastric Laparoscopic Band, Sleeve Gastrectomy, and Roux‐en‐Y Gastric Bypass (RYGB). A 1:2 propensity score match (PSM) was performed to match the bariatric surgery (BS) patients to non‐bariatric surgery (NBS) using age, gender, race, and baseline BMI. Patients were included if they had pre‐ and post‐operative sleep study data collected from in‐lab polysomnograms (PSG) or at‐home sleep studies (HST). The 4% desaturation rule for scoring hypopneas was used. Patients younger than the age of 18 were excluded from the study.

### 2.2 Outcome Measures

Basic demographics including age, sex, and race were obtained. Outcomes for OSA severity included both objective and subjective measures that were collected preoperatively closest to the time of surgery and postoperatively at the most recent follow‐up. Objective measures obtained from sleep studies included the AHI and SaO2 nadir, using full night polysomnograms (PSG) if available [20, 21]. 69% of patients had preoperative PSG data, and the remainder had home sleep apnea tests (HSTs). 29% of patients had postoperative PSG data available. Treatment response was measured using the modified Sher15 criteria, defined as a ≥ 50% reduction in AHI to a level of ≤ 15 events/h [22]. The Epworth Sleepiness Scale (ESS), a subjective patient‐reported measure of daytime sleepiness, was collected pre‐ and post‐operatively. BMI at baseline and postoperative time points was also reported [23].

### 2.3 Statistical Analysis

Mean (standard deviation) was used to present numeric variables and frequency (proportion) was used for categorical variables. Basic descriptive statistics were reported on the cohort as a whole and compared between the BS and NBS cohorts. AHI, ESS, SpO2 nadir, and BMI were compared at preoperative and postoperative time points. Sher15 treatment response was assessed and compared between cohorts. Non‐parametric t‐tests were used to analyze continuous variables, and [2] test was used for categorical variables. A multivariate linear regression adjusting for basic demographics and baseline metrics associated with undergoing BS was performed to identify independent predictors of the reduction in AHI from baseline following sleep surgery. Statistical significance was determined using an alpha level of 0.05 for all tests. All analyses were performed using RStudio Version 2023.03.0.

## 3 Results

### 3.1 Demographics and Baseline Characteristics

Following PSM, 72 patients (mean age: 61.33 years; 62% male; 83% White) were included in our study, of which 24 (33%) underwent previous BS and 48 (67%) did not (nBS) (Table 1). The distribution of basic demographics including age, sex, and race was similar between cohorts. The average time from surgery to the postoperative sleep study was 9.83 months (SD = 11.90) across the cohort and was similar between BS and nBS cohorts. The average time from bariatric surgery to HGNS was 128.06 months (SD = 84.82).

**TABLE 1:** Demographics and baseline characteristics.

Characteristic	*N*	Overall, *N* = 72^a^	Previous bariatric surgery, *N* = 24^a^	No previous bariatric surgery, *N* = 48^a^	*p* ^b^
Sex	72				> 0.9
Female		27 (38%)	9 (38%)	18 (38%)	
Male		45 (62%)	15 (62%)	30 (62%)	
Race	72				0.7
Black or African American		12 (17%)	3 (12%)	9 (19%)	
White		60 (83%)	21 (88%)	39 (81%)	
Age	72	61.33 (11.27)	62.75 (11.47)	60.62 (11.21)	0.5
Pre‐op BMI	72	30.26 (4.13)	31.18 (4.05)	29.80 (4.13)	0.2
Pre‐op AHI	72	35.24 (17.02)	37.29 (15.88)	34.21 (17.63)	0.4
Pre‐op ESS	69	10.20 (5.65)	11.57 (5.83)	9.60 (5.53)	0.14
Pre‐op SpO2 nadir	71	78.92 (7.50)	77.83 (9.28)	79.44 (6.52)	0.6
Pre‐op sleep study type	72				0.2
HST		22 (31%)	5 (21%)	17 (35%)	
PSG		50 (69%)	19 (79%)	31 (65%)	
Post‐op sleep study type	72				0.6
HST		51 (71%)	16 (67%)	35 (73%)	
PSG		21 (29%)	8 (33%)	13 (27%)	
Time to post‐op sleep study (months)	72	9.83 (11.90)	10.08 (11.83)	9.71 (12.06)	0.2

^a^ *n* (%); Mean (SD).

^b^ Pearson's Chi‐squared test; Fisher's exact test; Wilcoxon rank sum test.

The average preoperative AHI was 35.24 ± 17.02 events/h in the BS group and 34.21 ± 17.63 in the NBS group (*p* = 0.4). Pre‐treatment SpO2 nadir was 77.83% for BS and 79.44% for NBS (*p* = 0.6), while pre‐operative ESS was 11.57 for BS and 9.60 for NBS (*p* = 0.14).

### 3.2 Treatment Outcomes Following Sleep Surgery

Treatment response, defined by the modified Sher15 criteria, was achieved in 71% of BS patients and 56% of NBS patients (*p* = 0.2; Table 2). Patients in the BS group experienced a greater reduction in AHI compared to the NBS group (−28.44 ± 16.39 vs. −17.68 ± 21.23; *p* = 0.009). Post‐operative AHI was also lower in the BS group (8.85 ± 7.26 vs. 16.53 ± 17.39; *p* = 0.076), although this was not statistically significant. Following treatment, 46% of patients in the BS group returned to normal AHI (< 5 events/h), compared to 19% of patients in the NBS group. Additionally, none of the BS patients had severe residual OSA (AHI ≥ 30), whereas 12% of NBS patients remained in the severe range post‐operatively. The overall distribution of post‐operative OSA severity differed between groups, although the trend was not statistically significant (*p* = 0.053).

Both groups had comparable reductions in ESS (−4.67 vs. −3.44; *p* = 0.2), with a mean post‐operative ESS of 6.78 in BS and 6.17 in NBS (*p* = 0.5). The average post‐operative BMI was 30.77 ± 4.62 for BS and 29.59 ± 3.96 for NBS (*p* = 0.4).

**TABLE 2:** Comparing post‐surgical outcomes between patients with and without a history of previous bariatric surgery.

Characteristic	*N*	Overall, *N* = 72^a^	No previous bariatric surgery, *N* = 48^a^	Previous bariatric surgery, *N* = 24^a^	*p* ^b^
Post‐op AHI	72	13.97 (15.18)	16.53 (17.39)	8.85 (7.26)	0.076
AHI change	72	−21.27 (20.28)	−17.68 (21.23)	−28.44 (16.39)	0.009
Post‐op OSA Severity	72				0.053
Normal (AHI < 5)		20 (28%)	9 (19%)	11 (46%)	
Mild OSA (AHI 5–15)		30 (42%)	22 (46%)	8 (33%)	
Moderate OSA (AHI 15–30)		16 (22%)	11 (23%)	5 (21%)	
Severe OSA (AHI ≥ 30)		6 (8.3%)	6 (12%)	0 (0%)	
Post‐op ESS	71	6.37 (4.46)	6.17 (4.63)	6.78 (4.17)	0.5
ESS change	69	−3.81 (4.39)	−3.44 (4.50)	−4.67 (4.08)	0.2
Post‐op SpO2 nadir	72	83.06 (7.94)	82.86 (6.67)	83.48 (10.18)	0.2
Change in SpO2 nadir	71	4.16 (10.91)	3.42 (8.79)	5.72 (14.50)	0.4
Post‐op BMI	72	29.98 (4.20)	29.59 (3.96)	30.77 (4.62)	0.4
BMI change	72	−0.28 (2.71)	−0.22 (2.29)	−0.42 (3.46)	0.2
Sher15 treatment response	72	44 (61%)	27 (56%)	17 (71%)	0.2

^a^ Mean (SD); *n* (%).

^b^ Wilcoxon rank sum test; Fisher's exact test; Pearson's Chi‐squared test.

On multivariable linear regression adjusting for preoperative BMI, AHI, age, and sex, undergoing bariatric surgery remained independently associated with a greater reduction in AHI (*β* = −8.6; 95% CI: −16, −1.1; *p* = 0.026). Greater preoperative AHI was also independently associated with a greater reduction in AHI (*β* = −0.81; 95% CI: −1.0, −0.60; *p* < 0.001).

## 4 Discussion

Bariatric surgery is a well‐established treatment for morbid obesity and has been shown to improve both subjective and objective parameters of OSA [24, 25, 26]. However, a reduction in AHI and alleviation of subjective sleepiness following weight loss does not always imply complete resolution of OSA, as there remains a substantial group of patients with residual symptoms and disease burden that require further evaluation and treatment [27].

The mechanism of residual OSA following bariatric surgery is likely multifactorial, involving both anatomical and non‐anatomical factors. Craniofacial features such as reduced pharyngeal airway space, an inferiorly positioned hyoid bone, and narrow mandibular/maxillary skeletal structures may continue to compromise upper airway patency and are unaffected by weight loss [28]. Beyond anatomical factors, patients with persistent OSA following bariatric surgery may continue to have significant contributions from non‐anatomic factors such as poor muscle tone, low respiratory arousal threshold, and loop gain instability [29, 30, 31, 32]. Additionally, mounting evidence suggests that systemic inflammation plays a significant role in OSA pathogenesis in patients with obesity. Obesity is associated with chronic low‐grade systemic inflammation, driven by elevated levels of cytokines such as TNF‐α, IL‐6, and CRP [33, 34]. These mediators, released by visceral fat, impair upper airway neuromuscular control and ventilatory stability, increasing the risk of airway collapse. Intermittent hypoxia from OSA further amplifies inflammation, creating a feedback loop that exacerbates both conditions. Bariatric surgery has been shown to reduce systemic inflammation, including significant decreases in CRP and IL‐6, suggesting that at least some of these non‐anatomical contributors to OSA may be reversible [35, 36, 37]. However, whether these lingering effects on upper airway and ventilatory stability are fully reversible remains uncertain.

While CPAP is considered the first‐line therapy for residual OSA, studies have shown that bariatric surgery is associated with reduced CPAP compliance, often due to premature discontinuation of CPAP before achieving absolute remission [24]. This may be due to challenges faced by patients who had previously tried CPAP before undergoing bariatric surgery, such as difficulty obtaining a proper mask seal due to facial adiposity, hypoventilation from chest wall restriction, or intolerance to the higher positive airway pressures required for severe OSA [24, 38]. These negative experiences may reduce patients' interest in or willingness to use CPAP as a long‐term treatment for residual OSA after weight loss. While this population may benefit from enhanced counseling on the potential for improved CPAP tolerance post‐bariatric surgery, alternative treatments such as hypoglossal nerve stimulation (HGNS) should be considered for patients who remain CPAP‐intolerant or continue to experience OSA symptoms.

In our matched patient cohort, OSA patients who had previously undergone bariatric surgery demonstrated comparable Sher15 response rates, with a greater decline in AHI following HGNS therapy compared to patients who did not undergo bariatric surgery. Previous studies have demonstrated that compensatory upper‐airway muscle activation and responsiveness may be enhanced in overweight/obese patients without OSA, helping to maintain the patency of their highly collapsible airways and protect against OSA [39, 40]. While the same compensatory mechanism may have been activated in many patients prior to bariatric surgery due to significant weight gain, these mechanisms were previously insufficient to prevent airway collapse and OSA. Given that many long‐term physiological and anatomical adaptations to morbid obesity persist even after bariatric surgery, we hypothesize that the increased compensatory upper airway muscle activation/tone may persist, to a degree, in previously morbidly obese patients after weight loss following bariatric surgery [41, 42]. This inherent increase in pharyngeal dilator muscle tone may have an additive effect, enhancing the effectiveness of HGNS by decreasing upper airway collapsibility and providing a more stable airway environment, given previous findings suggesting that greater baseline pharyngeal dilator muscle compensation is associated with a more favorable response to HGNS therapy [43].

In addition, many patients undergoing bariatric surgery experience rapid weight loss that may lead to deeper anatomical changes, such as reductions in pharyngeal and tongue fat, which have been shown to correlate with improvements in OSA severity. A study by Wang et al. demonstrated that reductions in AHI following weight loss were largely mediated by the loss of tongue fat, highlighting the impact of adipose tissue reduction on upper airway patency [44]. Since HGNS works by stimulating the genioglossus muscle to protrude the tongue, its mechanical effectiveness may be amplified in patients with rapid fat loss and changes in tongue volume.

Although patients in our cohort who underwent bariatric surgery followed by HGNS experienced greater reductions in AHI, patient‐reported outcomes such as daytime sleepiness, measured by the ESS, did not differ significantly between groups. This highlights an important limitation of using AHI and Sher criteria as the sole measures of treatment success, as they may not fully capture the patient's subjective response to therapy. Nevertheless, both groups experienced a significant improvement in daytime sleepiness following treatment to a normalization of the ESS score (ESS ≤ 10) [17, 45]. This suggests that despite differing objective outcomes, both treatment pathways effectively and equally addressed daytime symptoms, which are often the primary complaint and major contributors to impaired quality of life in OSA [46].

Our findings suggest that bariatric surgery or medical weight loss may play an important role in patients who are initially contraindicated for an HGNS implant due to a BMI > 35 kg/m^2^, not only to meet eligibility criteria but also to potentially enhance responsiveness to HGNS therapy by optimizing both anatomic and physiologic conditions. A similar concept was proposed by Chang et al., who demonstrated that patients initially ineligible for HGNS due to complete concentric collapse (CCC) on DISE or severe OSA (AHI > 15 events/h) became eligible after undergoing multilevel surgery and subsequently achieved greater reductions in AHI and lower post‐treatment AHI levels compared to patients who underwent HGNS alone [47]. Although our study did not demonstrate significant differences in Sher15 response rates between patients with and without prior bariatric surgery, previous data from the ADHERE registry showed that while patients with BMI ≤ 32 and 32–35 kg/m^2^ demonstrated similar reductions in AHI with HGNS therapy, those with higher BMI were significantly less likely to meet Sher20 surgical success criteria, likely due to higher residual AHI [48]. This may reflect challenges in achieving adequate airway patency at comfortable HGNS settings in patients with increased airway collapsibility associated with elevated BMI—limitations that may be partially overcome through bariatric surgery in select cases. Given these considerations, the choice of therapy also warrants comparison. While CPAP is generally considered more effective at reducing AHI in controlled settings, its long‐term effectiveness is often limited by poor adherence. In contrast, HGNS may offer greater real‐world disease alleviation in select patients—particularly those with improved anatomical and physiological profiles after bariatric surgery—due to better tolerance. However, this hypothesis remains speculative and warrants further investigation.

Finally, while we support treating patients with residual OSA and symptoms after bariatric surgery, the decision to treat asymptomatic patients with residual OSA remains a complex issue. Previous large RCTs, including RICSSADA, SAVE, and ISAAC trials, did not demonstrate that CPAP treatment for OSA resulted in significant cardiovascular benefits [49, 50, 51]. However, these studies were limited by high residual AHI and poor CPAP compliance. Secondary analyses of these studies have suggested that increased CPAP compliance is associated with potentially better cardiovascular outcomes. It remains unknown whether similar outcomes would be observed for patients with moderate or severe OSA treated with HGNS or other devices, but there may be potential, as HGNS is generally better tolerated, leading to potentially greater disease alleviation compared to CPAP.

### 4.1 Limitations

There are important limitations of this study that must be considered, namely its retrospective design and limited sample size. The retrospective nature of this study increases the risk of selection bias based on the availability of data. For example, most patients who underwent bariatric surgery did so at a different institution and therefore lacked detailed information such as surgical indications, relevant comorbidities, and pre‐bariatric surgery AHI on the implanting institutions' EMR software. Moreover, our cohort likely overrepresents symptomatic post‐bariatric surgery patients with residual OSA who sought surgical intervention, potentially excluding asymptomatic individuals with similar OSA severity but who did not present to an ENT sleep surgery clinic for surgery evaluation. The relatively low sample size of patients who underwent bariatric surgery limits the power of our analysis and restricts the ability to draw causal relationships. Although we utilized PSM to control for potential confounders, other factors such as body composition, fat distribution (e.g., visceral vs. subcutaneous adiposity), severity and pattern of upper airway collapse, neuromuscular tone, arousal threshold, and systemic inflammation may influence surgical outcomes in the management of OSA. In addition, because this study relied on retrospective data, the type of sleep study (in‐lab PSG vs. HST) could not be standardized, and we were limited to the modalities available in the medical record. Given that HST tends to underestimate AHI compared to attended PSG, this limitation may have introduced a degree of misclassification of OSA severity in some patients. Lastly, while bariatric surgery patients may be more likely to have obesity hypoventilation syndrome (OHS), characterized by elevated CO_2_ and disproportionate nocturnal hypoxemia, these patients are generally not candidates for HGNS implantation due to their need for ventilatory support beyond the scope of HGNS therapy [52]. Although parameters like T90 and oxygen desaturation index (ODI) may indicate hypoventilation, definitive diagnosis requires arterial blood gas analysis or in‐lab PSG with CO_2_ monitoring, which were not uniformly available. Thus, undiagnosed OHS and its potential impact on outcomes cannot be fully assessed in this study.

### 4.2 Future Directions

Future studies should aim to employ a prospective design with a larger patient cohort to improve the reliability, accuracy, and completeness of data and provide more insight into this understudied relationship. Moreover, outcomes of OSA following bariatric surgery should eventually be compared to other weight loss modalities such as pharmacological treatment that have gained traction. The recently published SURMOUNT‐OSA trials evaluated tirzepatide, a dual glucagon‐like peptide‐1 (GLP‐1) and glucose‐dependent insulinotropic polypeptide (GIP) receptor agonist, in the management of moderate‐to‐severe OSA among patients receiving treatment with CPAP and those who were not [53, 54]. In both cohorts, tirzepatide resulted in a weight reduction of approximately 16%–17% and a reduction in AHI of 20–24 events per hour compared to placebo. An improvement in systolic blood pressure seen with tirzepatide was also observed among patients receiving CPAP therapy. However, considering the low (~50%) 1‐year adherence rates to GLP‐1 receptor agonists, there remains a significant emphasis on identifying treatment protocols that can optimize long‐term disease control [55].

Despite the significant reduction in AHI seen in patients who underwent bariatric surgery, differences in other clinically relevant endpoints remain to be seen. Future studies should aim to expand beyond AHI and ESS to assess subjective measures of OSA disease burden such as snoring and Functional Outcomes Sleep Questionnaire (FOSQ).

## 5 Conclusion

Our findings suggest that HGNS achieves comparable outcomes in patients who underwent previous bariatric surgery despite having a higher AHI and BMI at baseline. Persistent pharyngeal dilator muscle tone, more pliable redundant upper airway skin tissue, and pre‐selection bias for patients with non‐anatomical drivers for OSA are potential contributors to this observed trend. The potential benefits that may be derived from bariatric surgery in helping reduce OSA severity and making patients more amenable to HGNS require further study using more clinical endpoints of OSA.

## Conflicts of Interest

T.K. reports being a consultant for Inspire Medical and Nyxoah SA; he additionally receives Institutional Research Support from Cryosa Inc. T.K. is employed by the Veterans Affairs Medical Center, and the content herein does not represent the views of the United States Government. R.S. is a consultant for Cryosa Inc., XII Medical, and Inspire Medical Systems. Institutional research support from Inspire Medical systems. M.B. Chief Medical Officer for Nyxoah SA. C.H. has research support from Nyxoah SA and Inspire Medical. He has served as a consultant for Nyxoah SA, Inspire Medical, and Lunair. The other authors declare no conflicts of interest.

## Data Availability

The data that support the findings of this study are available on request from the corresponding author. The data are not publicly available due to privacy or ethical restrictions.

## References

[1] A. V. Benjafield , N. T. Ayas , P. R. Eastwood , et al., “Estimation of the Global Prevalence and Burden of Obstructive Sleep Apnoea: A Literature‐Based Analysis,” Lancet Respiratory Medicine 7, no. 8 (2019): 687–698, 10.1016/S2213-2600(19)30198-5. 31300334 PMC7007763

[2] W. T. McNicholas , M. R. Bonsigore , and Management Committee of ECAB , “Sleep Apnoea as an Independent Risk Factor for Cardiovascular Disease: Current Evidence, Basic Mechanisms and Research Priorities,” European Respiratory Journal 29, no. 1 (2007): 156–178, 10.1183/09031936.00027406. 17197482

[3] J. L. Chang , A. N. Goldberg , J. A. Alt , et al., “International Consensus Statement on Obstructive Sleep Apnea,” Int Forum Allergy Rhinol 13, no. 7 (2023): 1061–1482, 10.1002/alr.23079. 36068685 PMC10359192

[4] D. M. Hiestand , P. Britz , M. Goldman , and B. Phillips , “Prevalence of Symptoms and Risk of Sleep Apnea in the US Population: Results From the National Sleep Foundation Sleep in America 2005 Poll,” Chest 130, no. 3 (2006): 780–786, 10.1378/chest.130.3.780. 16963675

[5] S. Pannain and B. Mokhlesi , “Bariatric Surgery and Its Impact on Sleep Architecture, Sleep‐Disordered Breathing, and Metabolism,” Best Practice & Research. Clinical Endocrinology & Metabolism 24, no. 5 (2010): 745–761, 10.1016/j.beem.2010.07.007. 21112023

[6] P. E. Peppard , T. Young , J. H. Barnet , M. Palta , E. W. Hagen , and K. M. Hla , “Increased Prevalence of Sleep‐Disordered Breathing in Adults,” American Journal of Epidemiology 177, no. 9 (2013): 1006–1014, 10.1093/aje/kws342. 23589584 PMC3639722

[7] R. Rajala , M. Partinen , T. Sane , R. Pelkonen , K. Huikuri , and A. M. Seppalainen , “Obstructive Sleep Apnoea Syndrome in Morbidly Obese Patients,” Journal of Internal Medicine 230, no. 2 (1991): 125–129, 10.1111/j.1365-2796.1991.tb00419.x. 1865163

[8] T. Young , M. Palta , J. Dempsey , J. Skatrud , S. Weber , and S. Badr , “The Occurrence of Sleep‐Disordered Breathing Among Middle‐Aged Adults,” New England Journal of Medicine 328, no. 17 (1993): 1230–1235, 10.1056/NEJM199304293281704. 8464434

[9] H. Buchwald , Y. Avidor , E. Braunwald , et al., “Bariatric Surgery: A Systematic Review and Meta‐Analysis,” Journal of the American Medical Association 292, no. 14 (2004): 1724–1737, 10.1001/jama.292.14.1724. 15479938

[10] K. H. Saunders , L. I. Igel , and B. G. Tchang , “Surgical and Nonsurgical Weight Loss for Patients With Obstructive Sleep Apnea,” Otolaryngologic Clinics of North America 53, no. 3 (2020): 409–420, 10.1016/j.otc.2020.02.003. 32334866

[11] D. L. Greenburg , C. J. Lettieri , and A. H. Eliasson , “Effects of Surgical Weight Loss on Measures of Obstructive Sleep Apnea: A Meta‐Analysis,” American Journal of Medicine 122, no. 6 (2009): 535–542, 10.1016/j.amjmed.2008.10.037. 19486716

[12] K. Al Oweidat , A. A. Toubasi , R. B. A. Tawileh , H. B. A. Tawileh , and M. M. Hasuneh , “Bariatric Surgery and Obstructive Sleep Apnea: A Systematic Review and Meta‐Analysis,” Sleep & Breathing 27, no. 6 (2023): 2283–2294, 10.1007/s11325-023-02840-1. 37145243

[13] C. A. de Raaff , U. K. Coblijn , M. J. Ravesloot , et al., “Persistent Moderate or Severe Obstructive Sleep Apnea After Laparoscopic Roux‐En‐Y Gastric Bypass: Which Patients?,” Surgery for Obesity and Related Diseases 12, no. 10 (2016): 1866–1872, 10.1016/j.soard.2016.03.014. 27234342

[14] S. P. Patil , I. A. Ayappa , S. M. Caples , R. J. Kimoff , S. R. Patel , and C. G. Harrod , “Treatment of Adult Obstructive Sleep Apnea With Positive Airway Pressure: An American Academy of Sleep Medicine Clinical Practice Guideline,” Journal of Clinical Sleep Medicine 15, no. 2 (2019): 335–343, 10.5664/jcsm.7640. 30736887 PMC6374094

[15] T. E. Weaver and R. R. Grunstein , “Adherence to Continuous Positive Airway Pressure Therapy: The Challenge to Effective Treatment,” Proceedings of the American Thoracic Society 5, no. 2 (2008): 173–178, 10.1513/pats.200708-119MG. 18250209 PMC2645251

[16] T. E. Weaver and A. M. Sawyer , “Adherence to Continuous Positive Airway Pressure Treatment for Obstructive Sleep Apnoea: Implications for Future Interventions,” Indian Journal of Medical Research 131 (2010): 245–258. 20308750 PMC2972705

[17] P. J. Strollo, Jr. , R. J. Soose , J. T. Maurer , et al., “Upper‐Airway Stimulation for Obstructive Sleep Apnea,” New England Journal of Medicine 370, no. 2 (2014): 139–149, 10.1056/NEJMoa1308659. 24401051

[18] E. Thaler , R. Schwab , J. Maurer , et al., “Results of the ADHERE Upper Airway Stimulation Registry and Predictors of Therapy Efficacy,” Laryngoscope 130, no. 5 (2020): 1333–1338, 10.1002/lary.28286. 31520484 PMC7217178

[19] E. J. Kezirian , W. Hohenhorst , and N. de Vries , “Drug‐Induced Sleep Endoscopy: The VOTE Classification,” European Archives of Oto‐Rhino‐Laryngology 268, no. 8 (2011): 1233–1236, 10.1007/s00405-011-1633-8. 21614467

[20] T. M. Kaffenberger , E. M. Sina , B. Hambach , et al., “How We Measure Hypoglossal Nerve Stimulator Outcome Matters: Titration Versus Single Amplitude Efficacy Sleep Studies,” Journal of Clinical Sleep Medicine 21 (2024): 47–53, 10.5664/jcsm.11328. PMC11701276 39167433

[21] T. M. Kaffenberger , R. J. Soose , P. J. Strollo , M. Boon , and C. Huntley , “Classifying the Types of Therapeutic Sleep Studies,” Journal of Clinical Sleep Medicine 21, no. 7 (2025): 1321–1322, 10.5664/jcsm.11688. 40160007 PMC12225263

[22] A. E. Sher , K. B. Schechtman , and J. F. Piccirillo , “The Efficacy of Surgical Modifications of the Upper Airway in Adults With Obstructive Sleep Apnea Syndrome,” Sleep 19, no. 2 (1996): 156–177, 10.1093/sleep/19.2.156. 8855039

[23] M. T. Scharf , “Reliability and Efficacy of the Epworth Sleepiness Scale: Is There Still a Place for It?,” Nature and Science of Sleep 14 (2022): 2151–2156, 10.2147/NSS.S340950. PMC9759004 36536636

[24] P. Nastalek , K. Polok , N. Celejewska‐Wojcik , et al., “Impact of Bariatric Surgery on Obstructive Sleep Apnea Severity and Continuous Positive Airway Pressure Therapy Compliance‐Prospective Observational Study,” Scientific Reports 11, no. 1 (2021): 5003, 10.1038/s41598-021-84570-6. 33654165 PMC7925607

[25] P. Priyadarshini , V. P. Singh , S. Aggarwal , H. Garg , S. Sinha , and R. Guleria , “Impact of Bariatric Surgery on Obstructive Sleep Apnoea‐Hypopnea Syndrome in Morbidly Obese Patients,” Journal of Minimally Invasive Surgery 13, no. 4 (2017): 291–295, 10.4103/jmas.JMAS_5_17. PMC5607797 28872099

[26] K. Sarkhosh , N. J. Switzer , M. El‐Hadi , D. W. Birch , X. Shi , and S. Karmali , “The Impact of Bariatric Surgery on Obstructive Sleep Apnea: A Systematic Review,” Obesity Surgery 23, no. 3 (2013): 414–423, 10.1007/s11695-012-0862-2. 23299507

[27] C. J. Lettieri , A. H. Eliasson , and D. L. Greenburg , “Persistence of Obstructive Sleep Apnea After Surgical Weight Loss,” Journal of Clinical Sleep Medicine 4, no. 4 (2008): 333–338. 18763424 PMC2542489

[28] B. C. Neelapu , O. P. Kharbanda , H. K. Sardana , et al., “Craniofacial and Upper Airway Morphology in Adult Obstructive Sleep Apnea Patients: A Systematic Review and Meta‐Analysis of Cephalometric Studies,” Sleep Medicine Reviews 31 (2017): 79–90, 10.1016/j.smrv.2016.01.007. 27039222

[29] S. Javaheri and M. S. Badr , “Central Sleep Apnea: Pathophysiologic Classification,” Sleep 46, no. 3 (2023): zsac113, 10.1093/sleep/zsac113. 35551411 PMC9995798

[30] A. S. Jordan , D. G. McSharry , and A. Malhotra , “Adult Obstructive Sleep Apnoea,” Lancet 383, no. 9918 (2014): 736–747, 10.1016/S0140-6736(13)60734-5. 23910433 PMC3909558

[31] C. M. Sung , S. N. Tan , M. H. Shin , et al., “The Site of Airway Collapse in Sleep Apnea, Its Associations With Disease Severity and Obesity, and Implications for Mechanical Interventions,” American Journal of Respiratory and Critical Care Medicine 204, no. 1 (2021): 103–106, 10.1164/rccm.202011-4266LE. 33826879 PMC8437113

[32] O. M. Vanderveken , J. T. Maurer , W. Hohenhorst , et al., “Evaluation of Drug‐Induced Sleep Endoscopy as a Patient Selection Tool for Implanted Upper Airway Stimulation for Obstructive Sleep Apnea,” Journal of Clinical Sleep Medicine 9, no. 5 (2013): 433–438, 10.5664/jcsm.2658. 23674933 PMC3629316

[33] R. Mehra and S. Redline , “Sleep Apnea: A Proinflammatory Disorder That Coaggregates With Obesity,” Journal of Allergy and Clinical Immunology 121, no. 5 (2008): 1096–1102, 10.1016/j.jaci.2008.04.002. 18466782 PMC2720266

[34] T. Yokoe , K. Minoguchi , H. Matsuo , et al., “Elevated Levels of C‐Reactive Protein and Interleukin‐6 in Patients With Obstructive Sleep Apnea Syndrome Are Decreased by Nasal Continuous Positive Airway Pressure,” Circulation 107, no. 8 (2003): 1129–1134, 10.1161/01.cir.0000052627.99976.18. 12615790

[35] Z. Goktas , N. Moustaid‐Moussa , C. L. Shen , M. Boylan , H. Mo , and S. Wang , “Effects of Bariatric Surgery on Adipokine‐Induced Inflammation and Insulin Resistance,” Frontiers in Endocrinology 4 (2013): 69, 10.3389/fendo.2013.00069. 23772224 PMC3677351

[36] F. Illan‐Gomez , M. Gonzalvez‐Ortega , I. Orea‐Soler , et al., “Obesity and Inflammation: Change in Adiponectin, C‐Reactive Protein, Tumour Necrosis Factor‐Alpha and Interleukin‐6 After Bariatric Surgery,” Obesity Surgery 22, no. 6 (2012): 950–955, 10.1007/s11695-012-0643-y. 22527592

[37] S. M. Zagorski , N. N. Papa , and M. H. Chung , “The Effect of Weight Loss After Gastric Bypass on C‐Reactive Protein Levels,” Surgery for Obesity and Related Diseases 1, no. 2 (2005): 81–85, 10.1016/j.soard.2005.01.001. 16925219

[38] S. L. van Veldhuisen , M. F. van Boxel , M. J. Wiezer , et al., “Evaluation of CPAP Adherence in Bariatric Patients Diagnosed With Obstructive Sleep Apnea: Outcomes of a Multicenter Cohort Study,” Sleep & Breathing 27, no. 2 (2023): 535–544, 10.1007/s11325-022-02643-w. 35619018 PMC9135574

[39] J. Huang , S. J. Pinto , H. Yuan , et al., “Upper Airway Collapsibility and Genioglossus Activity in Adolescents During Sleep,” Sleep 35, no. 10 (2012): 1345–1352, 10.5665/sleep.2110. 23024432 PMC3443760

[40] S. A. Sands , D. J. Eckert , A. S. Jordan , et al., “Enhanced Upper‐Airway Muscle Responsiveness Is a Distinct Feature of Overweight/Obese Individuals Without Sleep Apnea,” American Journal of Respiratory and Critical Care Medicine 190, no. 8 (2014): 930–937, 10.1164/rccm.201404-0783OC. 25191791 PMC4299579

[41] M. L. Kendrick and G. F. Dakin , “Surgical Approaches to Obesity,” Mayo Clinic Proceedings 81, no. 10 (2006): S18–S24, 10.1016/s0025-6196(11)61177-4. 17036575

[42] P. Sumithran , L. A. Prendergast , E. Delbridge , et al., “Long‐Term Persistence of Hormonal Adaptations to Weight Loss,” New England Journal of Medicine 365, no. 17 (2011): 1597–1604, 10.1056/NEJMoa1105816. 22029981

[43] S. Op de Beeck , A. Wellman , M. Dieltjens , et al., “Endotypic Mechanisms of Successful Hypoglossal Nerve Stimulation for Obstructive Sleep Apnea,” American Journal of Respiratory and Critical Care Medicine 203, no. 6 (2021): 746–755, 10.1164/rccm.202006-2176OC. 32970962 PMC7958511

[44] S. H. Wang , B. T. Keenan , A. Wiemken , et al., “Effect of Weight Loss on Upper Airway Anatomy and the Apnea‐Hypopnea Index. The Importance of Tongue Fat,” American Journal of Respiratory and Critical Care Medicine 201, no. 6 (2020): 718–727, 10.1164/rccm.201903-0692OC. 31918559 PMC7068828

[45] M. Boon , C. Huntley , A. Steffen , et al., “Upper Airway Stimulation for Obstructive Sleep Apnea: Results From the ADHERE Registry,” Otolaryngology and Head and Neck Surgery 159, no. 2 (2018): 379–385, 10.1177/0194599818764896. 29557280

[46] M. W. Johns , “A New Method for Measuring Daytime Sleepiness: The Epworth Sleepiness Scale,” Sleep 14, no. 6 (1991): 540–545, 10.1093/sleep/14.6.540. 1798888

[47] C. P. Chang , S. Poomkonsarn , H. Giannakopoulos , Y. Ma , R. Riley , and S. Y. Liu , “Comparative Efficacy of Obstructive Sleep Apnea Patients Undergoing Multilevel Surgery Followed by Upper Airway Stimulation Versus Isolated Upper Airway Stimulation,” Journal of Oral and Maxillofacial Surgery 81, no. 5 (2023): 557–565, 10.1016/j.joms.2022.11.015. 36539190

[48] M. V. Suurna , A. Steffen , M. Boon , et al., “Impact of Body Mass Index and Discomfort on Upper Airway Stimulation: ADHERE Registry 2020 Update,” Laryngoscope 131, no. 11 (2021): 2616–2624, 10.1002/lary.29755. 34626128

[49] B. Balcan , E. Thunstrom , P. J. Strollo, Jr. , and Y. Peker , “Continuous Positive Airway Pressure Treatment and Depression in Adults With Coronary Artery Disease and Nonsleepy Obstructive Sleep Apnea. A Secondary Analysis of the RICCADSA Trial,” Annals of the American Thoracic Society 16, no. 1 (2019): 62–70, 10.1513/AnnalsATS.201803-174OC. 30130421

[50] R. D. McEvoy , N. A. Antic , E. Heeley , et al., “CPAP for Prevention of Cardiovascular Events in Obstructive Sleep Apnea,” New England Journal of Medicine 375, no. 10 (2016): 919–931, 10.1056/NEJMoa1606599. 27571048

[51] M. Sanchez‐de‐la‐Torre , A. Sanchez‐de‐la‐Torre , S. Bertran , et al., “Effect of Obstructive Sleep Apnoea and Its Treatment With Continuous Positive Airway Pressure on the Prevalence of Cardiovascular Events in Patients With Acute Coronary Syndrome (ISAACC Study): A Randomised Controlled Trial,” Lancet Respiratory Medicine 8, no. 4 (2020): 359–367, 10.1016/S2213-2600(19)30271-1. 31839558

[52] B. Mokhlesi , J. F. Masa , J. L. Brozek , et al., “Evaluation and Management of Obesity Hypoventilation Syndrome. An Official American Thoracic Society Clinical Practice Guideline,” American Journal of Respiratory and Critical Care Medicine 200, no. 3 (2019): e6–e24, 10.1164/rccm.201905-1071ST. 31368798 PMC6680300

[53] A. Malhotra , R. R. Grunstein , I. Fietze , et al., “Tirzepatide for the Treatment of Obstructive Sleep Apnea and Obesity,” New England Journal of Medicine 391, no. 13 (2024): 1193–1205, 10.1056/NEJMoa2404881. 38912654 PMC11598664

[54] S. R. Patel , “Entering a New Era in Sleep‐Apnea Treatment,” New England Journal of Medicine 391, no. 13 (2024): 1248–1249, 10.1056/NEJMe2407117. 38912659

[55] D. Do , T. Lee , S. K. Peasah , C. B. Good , A. Inneh , and U. Patel , “GLP‐1 Receptor Agonist Discontinuation Among Patients With Obesity and/or Type 2 Diabetes,” JAMA Network Open 7, no. 5 (2024): e2413172, 10.1001/jamanetworkopen.2024.13172. 38787563 PMC11127113

